# An Automatic Platform Based on Nanostructured Microfluidic Chip for Isolating and Identification of Circulating Tumor Cells

**DOI:** 10.3390/mi12050473

**Published:** 2021-04-21

**Authors:** Hei-Jen Jou, Li-Yun Chou, Wen-Chun Chang, Hsin-Cheng Ho, Wan-Ting Zhang, Pei-Ying Ling, Ko-Hsin Tsai, Szu-Hua Chen, Tze-Ho Chen, Pei-Hsuan Lo, Ming Chen, Heng-Tung Hsu

**Affiliations:** 1 Departments of Obstetrics and Gynecology, Taiwan Adventist Hospital, Taipei 105, Taiwan; peiyingling03@gmail.com (P.-Y.L.); 150188@tahsda.org.tw (K.-H.T.); pkmmcl@gmail.com (P.-H.L.); 2 Departments of Obstetrics and Gynecology, National Taiwan University Hospital, Taipei 100, Taiwan; 143178@tahsda.org.tw (L.-Y.C.); dtobgya1@yahoo.com.tw (W.-C.C.); mingchenmd@gmail.com (M.C.); 3 International College of Semiconductor Technology, National Yang Ming Chiao Tung University, Hsinchu 30010, Taiwan; neoho.icst07g@nctu.edu.tw; 4 School of Nursing, National Taipei University of Nursing and Health Science, Taipei 112, Taiwan; 5 Cytoaurora Biotechnologies Inc., Hsinchu 302, Taiwan; tina.zhang@cytoaurora.com; 6 Circulating Rare Cell Laboratory, Taiwan Adventist Hospital, Taipei 100, Taiwan; szu2520@gmail.com; 7 Department of Genomic Medicine and Center for Medical Genetics, Changhua Christian Hospital, Changhua 526, Taiwan; 46305@cch.org.tw; 8 Department of Genomic Science and Technology, Changhua Christian Hospital Healthcare System, Changhua 526, Taiwan; 9 Department of Obstetrics and Gynecology, Changhua Christian Hospital, Changhua 526, Taiwan

**Keywords:** circulating tumor cells, epithelial ovarian cancer, liquid biopsy, CD13, SKOV3

## Abstract

Circulating tumor cell (CTC) test is currently used as a biomarker in cancer treatment. Unfortunately, the poor reproducibility and limited sensitivity with the CTC detection have limited its potential impact on clinical application. A reliable automated CTC detection system is therefore needed. We have designed an automated microfluidic chip-based CTC detection system and hypothesize this novel system can reliably detect CTC from clinical specimens. SKOV3 ovarian cancer cell line was used first to test the reliability of our system. Ten healthy volunteers, 5 patients with benign ovarian tumors, and 8 patients with epithelial ovarian cancer (EOC) were recruited to validate the CTC capturing efficacy in the peripheral blood. The capture rates for spiking test in SKOV3 cells were 48.3% and 89.6% by using anti-EpCAM antibody alone and a combination of anti-EpCAM antibody and anti-N-cadherin antibody, respectively. The system was sensitive to detection of low cell count and showed a linear relationship with the cell counts in our test range. The sensitivity and specificity were 62.5% and 100% when CTC was used as a biomarker for EOC. Our results demonstrated that this automatic CTC platform has a high capture rate and is feasible for detection of CTCs in EOC.

## 1. Introduction

Despite recent advances in modern medicine, we are still facing many challenges in cancer diagnosis and treatment. Tissue biopsy is the only confirmatory diagnostic method for suspicious lesions while invasive exploratory surgery is required for cancer staging. Tissue biopsy has its own limitations due to its invasiveness and the fact that some lesions are in difficult-to-reach areas. In addition, tissue biopsy cannot assess the risk of cancer metastasis, the progression of the cancer, and the response to treatment [1]. Furthermore, it is very difficult to detect small metastatic lesions or minimal residual diseases using even the most advanced imaging technology [2]. Therefore, there is an urgent need to develop more precise methods to facilitate screening, diagnosis, and treatment of cancers.

CTCs are the cells shed from the primary site of the tumor that then entered the peripheral blood circulation, which has the potential to develop into new tumor foci [3]. Although many steps involved in cancer metastasis are still unclear, CTCs obviously play a critical role in cancer metastasis [4,5]. We can repeatedly take the patient’s CTCs as a “real-time liquid biopsy” to predict cancer recurrence, monitor the effect of treatment, and understand potential drug resistance mechanisms [6]. It is estimated that there are 1–10 CTCs per mL of whole blood in the peripheral blood of patients with metastatic cancer [7]. Because of the scarcity of CTCs in the blood, it not only is technically difficult to capture CTCs from the blood of cancer patients but also requires a lot of blood for the test.

Since 1990, there have been many technological developments and applications to enrich and identify CTCs. However, even though considerable progress has been achieved in recent years, the CTC detection technology is still not widely adopted for clinical practice because of its limitations including the scarcity and fragility of CTCs, the heterogeneity of CTCs, the lack of standard detection methods, and the need for sensitivity and specificity [8]. Most laboratories detect CTC manually which has low interrater reliability and low throughput. Therefore, the key point to overcome the current limitations is to develop an automated CTC detection technology providing unbiased result and high sensitivity will benefit clinicians taking care of the cancer patients. The rapidly improving microfluidic-based technology over the last ten years can be a sensitive, efficient, and fully automated system with a portable size [8,9].

Ovarian cancer is a common cancer in women with extremely poor prognosis. With the current diagnostic techniques, more than half of ovarian cancers are diagnosed at advanced stage [10]. Therefore, there is an urgent need for new diagnostic tools to assist in the management of ovarian cancer, including early diagnosis, evaluation before and after surgery, prognostic judgment, detection of minimal residual disease or early recurrence, assessment of treatment response, and even drug selection. The purpose of this study is to evaluate the applicability of a newly designed, fully automated microfluidic CTC platform, in the clinical evaluation of ovarian cancer.

In the present study, we used an automatic microfluidic immunoaffinity-based system to achieve reliable reproducibility with high throughput. This automated system consists of several parts, including a nano-structured microfluidic biochip, an automatic CTC enrichment and staining system, and an automatic CTC scanning and locating system. The capture efficiency of the automatic system was tested by spiking tests of SKOV3 ovarian cancer cell line. A preliminary test for clinical feasibility on epithelial ovarian cancer (EOC) from consented human volunteers was also conducted.

## 2. Methods

### 2.1. Cell Line Preparation

A human ovarian adenocarcinoma cell line, SKOV3 (ATCC^®^ HTB-77TM, Manassas, VA, US), was used for the cell spiking test. The SKOV3 cells were cultured in McCoy’s 5A medium (BioConcept, Allschwil, Switzerland), supplemented with 10% fetal bovine serum (FBS), 100 units/mL penicillin (Gibco, Grand Island, NY, USA). Peripheral blood mononuclear cells (PBMCs) were separated from the whole blood of healthy volunteers by a density gradient centrifugation method.

Two different enrichment strategies were used for spiking test, including 1:40 biotinylated anti-EpCAM antibody only, and an antibody cocktail combined with 1:40 biotinylated anti-EpCAM antibody and 0.005 mg/mL biotinylated anti-N-cadherin antibody (Combi). Prior to their mixture, both SKOV3 cells and PBMCs were incubated with anti-EpCAM antibody or Combi cocktail at 37 °C for 45 min in a 15 mL centrifuge tube. Then 3 mL DPBS was added to each tube and the tubes were centrifuged at 300× *g* for 10 min to collect the cell pallets and remove free antibodies. The cell mixture for spiking test was prepared by spiking 5 × 10^3^ SKOV3 cells into PBMCs of 2 mL whole blood origin in 200 μL of DPBS. The SKOV3 cell number was counted using Countess ^TM^ II FL Automated Cell Counter (ThermoFisher, Waltham, MA, USA).

### 2.2. Microfluid Chip

**Figure 1:**
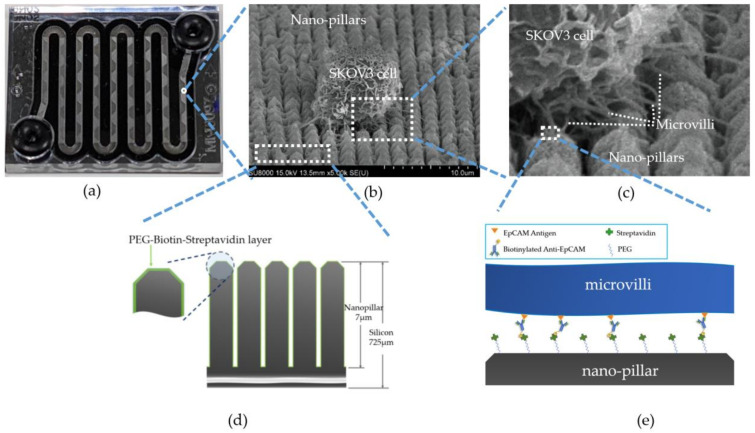
The V-BioChip. (**a**) The silicon-based microfluidic V-BioChip. (**b**) An intact SKOV3 cell is captured by the V-BioChip (under 5000× scanning electron micrography). (**c**) The microvilli of SKOV3 cells are firmly attached to the surface of the nano-pillars of the V-BioChip. (**d**) The illustration of nano-pillars (lateral view). The surface of the nano-pillars was covered a thin PEG-biotin-streptavidin layer. The head of each nano-pillar was modified like a volcano cone. (**e**) The chip captures the CTCs via the interaction between the PEG-biotin-streptavidin layer on the nano-pillars and the biotinylated antibody on the microvilli of the CTCs.

The V-BioChip (CytoAurora Inc., HsinChu, Taiwan), with a size of 32 × 34 × 0.7 mm, is a silicon-based chip with nano-pillar arrays on the inside of microfluidic chambers (Figure 1). Nanotexturing on the chips can improve CTC adherence relative to a flat surface [11]. The structure and production process of the predecessor of V-BioChip, Cral Chip, has been described in detail in Ma’s report [12]. To improve the chip’s capture efficiency, we modified original design of Coral Chip to adjust the distance between the nano-pillars on the microfluidic chip and the shape of the nano-pillars. In brief, metal-assisted chemical etching (MACE) technology was used to form matrix-arranged nano-pillars on the chip surface. The tip of the pillars is modified into a shape of volcanic cone to increase the contact surface between the microvilli of the target cells and the nano-pillars. The pretest of the chips revealed that too wide a groove distance may make the cells sink into the groove and distort the cells, which causes difficulties in subsequent immunofluorescence staining and cell identification. Thereafter, the polyethylene glycol-biotin (PEG-biotin) layer was modified on the surface of the wafer by vapor deposition method. Streptavidin, a tetrameric protein with high binding affinity to biotin [13], was then attached to the biotin end of the PEG-biotin using liquid deposition technology. The streptavidin–biotin interaction is one of the strongest non-covalent biological interactions currently known and can markedly increase the capture efficiency to the target cells [14,15]. When the mixed cell suspension flows over the chip, the target cells will be captured by the V-BioChip by the interaction between PEG-biotin-streptavidin layer on the nano-pillars and the biotinylated antibody on the microvilli of CTCs (Figure 1), and most other cells will be washed away.

### 2.3. Cell Spiking Test

**Figure 2:**
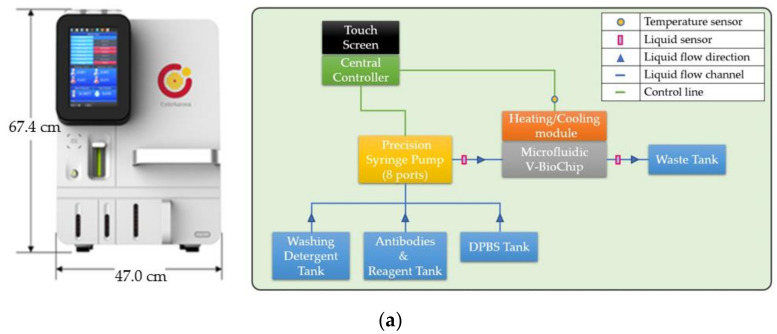

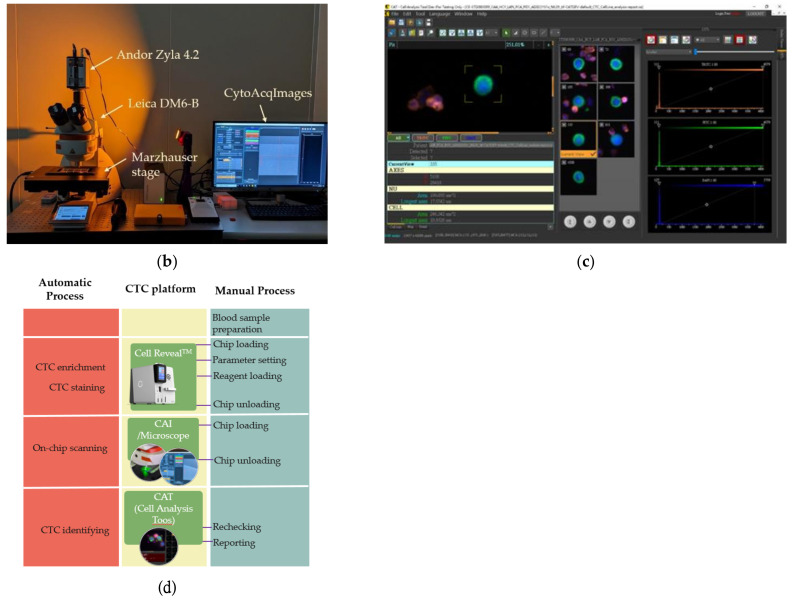
The automatic microfluidic-based CTC platform. (**a**) The outlook and modules of the Cell Reveal^TM^ system. After the chip and reagents being put in, the system can automatically carry out the enrichment and staining processes according to the pre-set condition. (**b**) The automatic scanning and locating system. This microscope is controlled by the CytoAcqImages system to perform automatic scanning of V-BioChip and positioning of target cells. (**c**) The CAT (Cell Analysis Tools) system, which can identify target cells according to the immunofluorescence staining on the cells by using pre-set parameters and deep learning AI function. (**d**) The schematic of the laboratory procedure.

The Cell Reveal^TM^ machine (CytoAurora Inc., HsinChu, Taiwan) was used for the enrichment and staining of the CTCs. Before the test, the V-Biochip was set up into the machine and various solutions (capture antibody, immunofluorescent staining solution, etc.) were put into the container in the machine (Figure 2a). After that, the mixed cell suspension of SKOV3 and PBMCs was injected into the Cell Reveal^TM^ system, and the system automatically processed all subsequent CTC enrichment and staining procedures. The inputted cell mixture was then fixed in 4% paraformaldehyde. Subsequently, 0.1% of Triton X-100 (ThermoFisher, Waltham, MA, USA) and 2% BSA (Bovine serum albumin) were added to increase the cellular permeability. The cell mixture passes through the V-BioChip at a flow rate of 0.6 mL/h, allowing the target cells to fully contact the chip to achieve an optimal capture rate. As the cell-rich concentrate flows through the microfluidic chips, the streptavidin on the chips captures the target cells bound with biotinylated anti-EpCAM antibodies. The process of CTC enrichment and staining were done overnight to achieve the best staining, but this process can be completed within four hours.

In order to distinguish CTCs from white blood cells, microfluidic chips were incubated with anti-EpCAM antibody (R&D Systems, Minneapolis, MN, USA) conjugated with FITC (for the detection of CTCs), as well as anti-CD45 (Agilent, Santa Clara, CA, USA) antibody conjugated with TRITC (for the detection of white blood cells) and 4′,6-diamidino-2-phenylindole (DAPI; Invitrogen, Carlsbad, CA, USA) (for the detection of nucleated cells). The CTC was defined as an EpCAM+/CD45-/DAPI+ intact cell.

After the enrichment and staining steps, V-BioChip is moved to the CTC scanning platform for further analysis. This state-of-the-art CTC scanning platform is composed three main parts, including a modified upright fluorescent microscope and two sets of self-developed software (CytoAcqImages system for automatic image scanning and CAT automatic cell identification system). The upright fluorescent microscope (Leica DM6 B, Leica Microsystems GmBH, Wetzlar, Germany) is equipped with Leica HC PL APO 10x/0.45 microscope objective, spectra III light engine (Lumencor, Beaverton, OR, USA) with wavelength range: 380 nm~750 nm, Andor Zyla 4.2 sCMOS camera and Marzhauser scanning stage for 4 slides and is controlled by the CytoAcqImages (CAI) system. The CAI system can be used with any brand of microscope, light controller, motorized XYZ stages and sCMOS camera (Figure 2b). 

Under the control of CAI system, the microscope automatically focuses and scans the V-BioChip, as well as activates the exposure with a fluorescent carousel. A fully automatic segmented photographing method was adopted to obtain high-resolution full-area images of V-BioChips. Then, the image files were montaged together to form a whole biochip TIFF image file.

V-BioChip is pre-installed with special positioning marks. During the manufacturing process of V-BioChip, several cross-alignment marks are etched on the chip outside the microfluidic channel. When the CAI system focuses on these marks, it also records the coordinate information to the full chip image file. When the image interpretation system reads the chip image file, it can use the coordinate information to calculate the specific position of the specific cell on the chip. In addition, this precise positioning function can also help to find specific cells on the chip again, which can assist in re-evaluating specific cells and even further single-cell analysis. 

**Figure 3:**
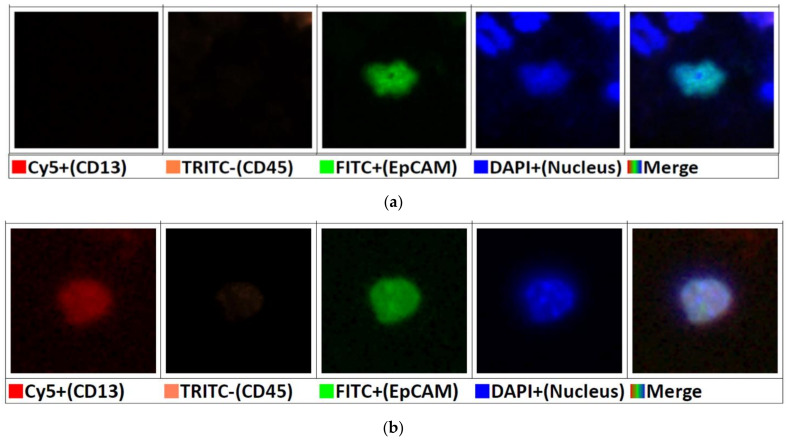
Immunofluorescence staining of circulating tumor cells and characterization with cancer stem cell specific molecular marker CD13: (**a**) CTC from a patient with endometrioma stained CD13-/EpCAM+/CD45-/DAPI+. The shape of nucleus seems to be distorted. Alternation in nuclear shape may be due to the cell condition (like during mitosis), in processes associated with cell death, the lab procedure, and condition of photographing. (**b**) CTC from an EOC patient (high-grade serous cystadenocarcinoma, FIGO stage Ic3) showed CD13+/EpCAM+/CD45-/DAPI+ staining.

The full chip image files were then transmitted to CAT system (Figure 2c). CAT system can identify target cells according to the immunofluorescence staining on the cells by using pre-set parameters and deep learning AI function. Count-in/filter-out criteria were used to identify the cells while EpCAM+/CD45-/DAPI+ for CTCs and EpCAM-/CD45+/DAPI+ for WBCs (Figure 3a). 

Figure 2d shows the schematic of the whole laboratory process. The integration of these automatic systems greatly reduces manual processes and improves the standardization and output of the CTC test, making the possibility of liberal clinical use one step forward.

Three separate spiking tests were performed for both the single ant-EpCAM antibody and Combi strategy. The results are expressed as the mean recovery rate ± standard deviation (SD).

### 2.4. Linearity between the Numbers of Captured Cells and Spiked Cells

The relationship between the captured cell numbers and the CTC numbers was analyzed by spiking SKOV3 cells into DPBS. We incubated 625 SKOV3 cells with 5 μL cocktail capture antibodies in 200 μL DPBS at 37 °C for 45 min and then centrifuged with 400 g for 10 min. A serial of 2-fold dilutions was performed to allow for linear regression analysis of the estimated cell numbers being 625, 312, 156, 78, and 39. The diluted cell suspensions were then injected into Cell Reveal^TM^ System to capture SKOV3 cells by streptavidin–coated chip with a flow speed of 0.6 μL/min. The captured cells were stained by EpCAM/CD45/DAPI on chip and subsequently were scanned and counted by the automatic CAT system. 

Each spiking cell number is tested twice. In addition to linear regression analysis, the recovery rate of each test was also calculated. The results of recovery rate were expressed as mean recovery rate ± SD.

### 2.5. Feasibility Study

In order to examine the clinical feasibility of the test, we recruited participants to join the study, including 10 healthy subjects without any type of ovarian tumor, 5 women with benign ovarian tumor, and 8 patients with epithelial ovarian cancer (EOC). FIGO criteria were used for the staging of EOC. The study has been approved by the IRB committee in the Taiwan Adventist Hospital and Changhua Christian Hospital. All methods were carried out in accordance with relevant guidelines and regulations, and each participant completed a written consent before they received the test. 

In this study, anti-CD13 antibody (Abcam, Cambridge, UK) was also used for ICC staining to identify CTCs that carry CD13 (Aminopeptidase N) surface marker. Previous studies have demonstrated that CD13 is a surface marker for semiquiescent CSCs, which is related to chemo/radiation resistance of the disease [16,17]. Both CD13 and EpCAM may express in SKOV3 cells and in ovarian cancers [18,19]. When an intact cell shows ICC staining of CD13+/EpCAM+/CD45-/DAPI+, it is defined as a CD13+ CTC (Figure 3b).

## 3. Experimental Results

The enrichment and staining processes were achieved within as short as 4 h in the Cell Reveal^TM^ system, but overnight handing will not change the readouts. The automatic on-chip scanning and cell locating by CAT took about 15 min. 

V-BioChip has excellent capture ability for target cells. Figure 1a demonstrates the gross appearance of a V-BioChip and the image of an SKOV3 cell captured by V-BioChip under a scanning electron microscope (SEM) at 5000× magnification (Figure 1b). It can be seen from the SEM image that there were many microvilli on the surface of the SKOV3 cell, and some microvilli firmly adhered to the nanopillars of V-BioChip (Figure 1c). The captured SKOV3 cells had intact morphology without distortion, which means that V-BioChip can capture the targeted cells under the automatic process without obvious damage.

**Figure 4:**
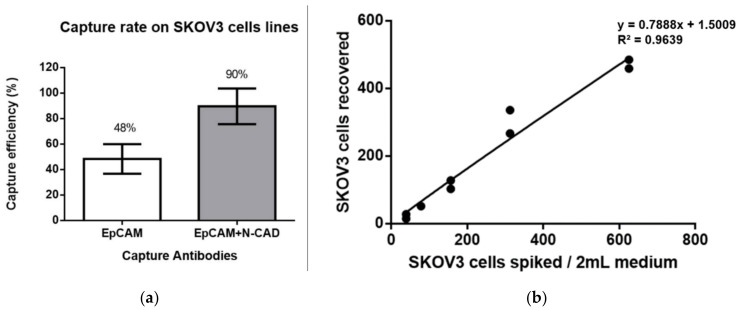
Capture efficiency of SKOV3 cells: (**a**) Capture efficiency of SKOV3 cells by using a single anti-EpCAM antibody or a combination of anti-EpCAM antibody and anti-N-cadherin antibody as capture strategy. The bar reveals the overall efficiency, defined as the target cell number counted by the automatic CAT system as a proportion of the spike cell number, in both PBMCs. CTC is defined as an intact EpCAM+/CT45-/DAPI+ cells. (**b**) The linearity of Cell Reveal^TM^ system in low spiking number by spiking SKOV3 cells in media. Linear regression was calculated between the number of the captured cells (*Y*-axis) and the number of spiked cells (*X*-axis).

Because of the scarcity of CTC, capture efficiency is critical for a successful CTC test platform. With EpCAM antibody alone as the capture antibody, the recovery rate was 48.3% with a SD of 11.7%. Considering the mesenchymal characteristics of SKOV3 cells, we used a mixture of an epithelial marker antibody (anti-EpCAM antibody) as well as an EMT marker antibody (anti-N-cadherin antibody) to make a capture antibody cocktail (Combi). The Combi strategy markedly increased the capture rate to 89.6% (SD: 11.8%) (Figure 4a).

The analysis of the linear relationship between the number of spiking cells and the number of captured cells at low spiking cell numbers is also an important indicator of the capture efficiency of the CTC platform. The present study showed a high recovery rate and a linear relationship even in low spiking cell numbers (39–625). The linear regression was y = 0.7888x + 1.5009 (R^2^ = 0.9639) with an average recovery rate of 73.5% (SD: 11.8%) (Figure 4b).

**Figure 5:**
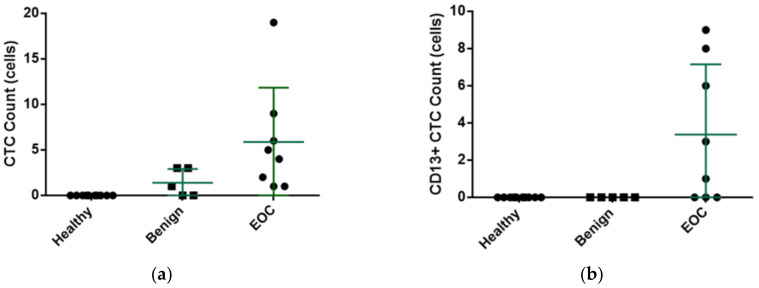
CTC counts and CD13+ CTC counts in healthy volunteers, patients of benign ovarian tumor and EOC patients: (**a**) No CTCs were detected in the peripheral blood of the ten healthy subjects. As for patients with benign ovarian tumors and EOC, the average peripheral blood CTCs were 1.40 (1.40 + 1.51, ranging 0–3) and 5.88 (5.88 + 5.96, ranging 1–19), respectively. (**b**) Neither healthy volunteers nor patients with benign ovarian tumors could detect CD13+ CTCs in the peripheral blood. The average number of CD13+ CTCs in the peripheral blood of EOC patients was 3.375/4 mL (ranging 0–19).

The results of clinical validity tests are summarized in Table 1 and Figure 5. No CTCs were detected in the peripheral blood of the ten healthy subjects. As for patients with benign ovarian tumors and EOC, the average peripheral blood CTCs were 1.40 (1.40 ±1.51, ranging 0–3) and 5.88 (5.88 ± 5.96, ranging 1–19), respectively (Figure 5a). If a cutoff of ≥4 CTCs in 4 mL of blood is used to screen for EOC, the sensitivity is 62.5% and the specificity is 100%, respectively.

Our results revealed that neither healthy subjects nor patients with benign ovarian tumors could detect CD13+ CTCs in the peripheral blood. The average number of CD13+ CTCs in the peripheral blood of EOC patients was 3.375/4 mL (ranging: 0–19) (Figure 5b). The results showed a sensitivity of 62.5% and a specificity of 100%, respectively, if we used the presence of CD13+ CTCs or not as the cutoff. Further analysis showed a sensitivity of 75% and a specificity of 100%, respectively, if we used the cutoff as ≥ 4 CTCs or the presence of CD13+ CTCs in 4 mL of blood.

## 4. Discussion

In the present study, we show a fully automated CTC detection platform that can efficiently capture, identify, scan, locate, enumerate, and even characterize CTCs on-chip. The development of fully automated systems has greatly increased the possibility of CTC testing being utilized in clinical practice. This study also preliminarily tested the validity of the platform for patients with EOC and found that it has considerable potential in the detection of CTCs in EOC patients and could assist the treatment decision in EOC and even other cancers. 

Due to the rarity of CTCs in the peripheral blood of cancer patients, high enrichment efficiency is critical for CTC detection platform. The design and manufacture of microfluidic chips plays an important role in the entire system. V-BioChip is an immunoaffinity-based microfluidic chip which is mainly based on the affinity binding between CTCs and the surface of the nanopillar on the chip. The surface of nanopillars is coated with a PEG-biotin-streptavidin layer and the target cells are pre-labeled with biotinylated monoclonal antibody. Nanotexturing improved the adhesion of microvilli and invadopodia of CTCs to the surface of nanopillars which was functionalized with streptavidin [12]. 

Epithelial cell adhesion molecule (EpCAM) is a cancer-related antigen. Cancer tissues of epithelial origin often overexpress EpCAM [20,21,22]. Therefore, in the past few decades, EpCAM has been used by many enrichment technologies as the main cell membrane marker to isolate CTCs, and considerable progress has been made in the use of EpCAM-based CTC detection technologies. However, there are many reasons why EpCAM-based technologies cannot effectively detect CTCs. The expression of EpCAM in variant cancers is quite different [23]. In addition, epithelial-to-mesenchymal transition (EMT) is a key step of the metastatic cascade. This process allows epithelial cancer cells to acquire some mesenchymal characteristics while losing epithelial cell phenotype, resulting in a decrease in the cell’s EpCAM expression [24,25]. For example, the expression of EpCAM in patients with ovarian cancer depends on their histological subtypes. Overall, 73% of ovarian cancers overexpress EpCAM, but serous ovarian cancers have a lower EpCAM overexpression rate (55%) [23].

Due to the downregulation of CTC EpCAM expression during the EMT process, the use of EpCAM-based enrichment techniques may not efficiently capture the CTC populations that are currently EMT [24,25,26]. SKOV3 is an EOC cell line with mesenchymal phenotype. It is reasonable that the present study shows a low capture efficiency of 48% for SKOV3 cells when using a single epithelial antibody as the capture antibody. For cancer cells undergoing EMT, further strategies must be taken to achieve better capture efficiency.

The combined use of epidermal and mesenchymal markers not only increases the probability of capturing CTCs of different phenotypes but also reduces the omission of EpCAM-negative CTCs. Po and his colleagues demonstrated that adding anti-N-cadherin antibodies to anti-EpCAM antibodies can increase the efficiency of capturing CTCs when using immunomagnetic beads as a strategy for isolating CTCs [27]. Another study showed that using five different types of antibodies on the CTC platform can offer a significant improvement in cell-isolation efficiency, even from tiny amounts of blood (250 μL–1 mL) or in non-metastatic breast cancer [28]. The present research also reached similar conclusion as the previous studies. Using the Combi strategy by mixing an epithelial antibody and a mesenchymal antibody as the enrichment antibody combination can significantly overcome the adverse effect of downregulation of EpCAM expression caused by EMT on the CTC capture efficiency of the microfluidic system and achieve an optimal capture rate. Most studies were conducted by using PBMCs suspensions of cancer cells that mimic actual conditions. The donor’s blood can interact with the spiked cells and markedly affect the recovery efficiency. Our results show that the automated CTC platform combined with V-BioChip and Cell Reveal ^TM^ system can achieve excellent CTC capture efficiency of 90% by using Combi strategy. Even in low cell count, the system also demonstrated excellent capture efficiency and device linearity with R^2^ of 96%.

Although the Combi strategy can increase the capture rate of CTCs, Po et al. reported that this method may cause false positives due to the simultaneous capture of circulating endothelial cells (CECs). They use vascular endothelial-cadherin co-staining to distinguish CECs from CTCs and therefore reduce the false positive [27]. In the present study, stem cell specific antibody (anti-CD13 antibody) was used to co-stain the cells, and the preliminary results show that it may also eliminate the “false positive”. However, further investigation is still needed to get a solid conclusion for the limited cases in the present study. 

Current methods for CTCs identification and analysis depend on immunofluorescence technologies and fluorescence microscopic imaging by characterizing the tumor cells with specific markers. Up until now, most of the microfluidic chip-based CTCs platforms still rely on manual operations for cell screening and interpretation after immunofluorescent staining of target cells, which is time-consuming and causes inconsistent results. So far, only a limited number of automated CTCs microscope systems have been reported [29]. The automated CTCs scanning and identification system can complete the scanning of the target cells in a short time, which not only saves time and manpower but also helps to establish standard processes. Our state-of-the-art automated microscope control system and CTCs scanning and identification system can automatically number and locate target cells in addition to automatic scanning and identification of target cells. Numbering and positioning functions can find specific target cells when manual inspection or re-evaluation is required. In addition, these functions can re-locate specific cells on the chip in a very short period of time, which is very useful when single cell analysis is required.

In the face of the extremely poor prognosis of ovarian cancer, CTC has the potential to provide more information beyond the current testing technology to assist treatment decisions. In the past decade, there have been many studies showing the use of CTCs as a “real-time liquid biopsy” for the diagnosis and treatment of ovarian cancer. However, these studies show a low positive rate of CTCs and a low median of CTCs [8,30,31,32]. The possible reason is the early onset of peritoneal dissemination in EOC, with only one third of the patient has distant metastases, leading to insufficient CTCs in the peripheral blood to be detected [33,34]. In addition, most of these studies do not report the sensitivity and specificity of the technology used [24,35,36,37]. Therefore, the clinical application of CTCs in management of EOC still needs further confirmation.

These studies involved different CTC technologies, including immunomegnatic enrichment, density-gradient separation followed by immunostaining or RT-PCR and microfluidic technology. The CellSearch ^TM^ system, approved by the FDA in 2004, represents the first-generation CTC test platform and has been used in the advanced stage cancers of breast, colorectal and prostate [38,39,40]. The reported detection rates of the CellSearch ^TM^ system varied between 14.4–40% [41,42,43,44,45] but might be as high as 60% in stage III-IV EOC [46]. These results revealed that the CellSearch ^TM^ system could not effectively detect EOC at early stage. In general, the density-gradient separation seems to surpass the immunomagnetic technology in detection rate. However, the detection rates of CTCs in EOC at early stage remain low. The diverse CTC test technologies and methodologies make it extremely difficult to compare the results between different studies. 

Recently, variant microfluidic chips have been introduced for CTC isolation in EOC. Rao and his colleagues reported a detection rate of 87% by using IsoFlux system, an immunomagnetic-based microfluidic CTC platform [37]. A drawback of the IsoFlux system is that the isolation of CTCs depends solely on expression of EpCAM and cytokeratins. Lee et al. reported a high detection rate up to 98.1% by using an immunoaffinity based microfluidic system without indicating the sensitivity and specificity of the test [36]. Guo et al. used a size-based microfluidic system to detect HE4+ CTCs in patients with suspicious ovarian cancer and showed a sensitivity of 73.3% and a specificity of 86.7% [47]. The identification of CTCs in the latter two studies still relied on manual operation, which is labor-intensive. At the present stage, it is difficult to judge whether the microfluidic technology can be effectively used as a superior technology as liquid biopsy for EOC. Although the results seem to be promising, the number of studies and cases are limited. However, our system seems to have the advantages of full automation and the ability to perform cell characterization on-chip. 

In addition to enumeration of CTCs, single-cell phenotypic characterization can provide additional biochemical information to assist clinical diagnosis and treatment decisions [19]. Aminopeptidase N (CD13) is a surface marker of cancer stem cells (CSC). It plays a role in cancer cell invasion and is also a candidate for treatment resistance, recurrence, and poor prognosis, which is highly expressed in early stage of EOC [48,49]. Van Hensbergen and his colleagues found that the expression of CD13 in EOC was associated with the histological subtype: CD13 expression in tumor cells was observed in 80% of the patients with a serous carcinoma and 100% of the patients with a mucinous carcinoma and in only 20% of the clear cell carcinoma patients [49,50]. To the best of our knowledge, this is the first attempt to isolate and identify CD13+ CTCs in EOC patients.

Although the case number in the present study is limited, there are several meaningful findings. First, no matter which cutoff is used, there is a high sensitivity, which can greatly simplify the interpretation of clinicians. Second, the positive rate of CD13+ CTCs in serous adenocarcinoma is consistent with the histological study by Van Hensbergen. Third, CTCs or CD13+ CTCs can be detected even in the early stage (stage I) EOC. It means that CTCs have appeared in the blood at least in some early EOCs, indicating the possibility of micrometastasis even in the early state of EOC by surgical staging. Finally, due to the full automation of the laboratory processes, it is technically possible to simultaneously detect one or more additional cell markers in. In other words, it has the potential to provide a comprehensive cell profiling for clinical applications, but more experimental validation is still needed.

## 5. Conclusions

In conclusion, as the advantage of microfluidic chips and automated systems, CTCs detection has become more and more efficient and accurate. Although many microfluidic-based methods have been developed in the past decade, most of them cannot be applied clinically due to lack of automation, low throughput, and high cost of microfluidic chips. This study shows that the automated Cell Reveal ^TM^ system has the potential for widespread clinical application to aid in the treatment decisions of EOC and even other cancers. However, in spite of the very promising preliminary results, more clinical experience is needed to reach further conclusions due to the complexity and heterogeneity of cancers.

## Figures and Tables

**Table 1:** Summary of CTC and CD13+ CTC counts in patients with benign ovarian tumor or epithelial ovarian cancer.

Case Number	Age	Diagnosis	Staging	Number of CTCs	Number of CD13+ CTCs
**Benign**					
1	45	Endometrioma	-	3	0
2	48	Endometrioma	-	1	0
3	42	Mature teratoma	-	0	0
4	32	Endometrioma	-	3	0
5	34	Endometrioma	-	0	0
**Malignant**					
1	56	High grade serous cystadenocarcinoma	I	6	6
2	56	High grade serous cystadenocarcinoma	I	1	1
3	21	Endometrioid carcinoma	I	5	0
4	61	Endometrioid carcinoma	I	19	8
5	67	High grade serous cystadenocarcinoma	II	2	0
6	60	Clear cell carcinoma	III	1	0
7	48	High grade serous cystadenocarcinoma	III	4	3
8	58	Endometrioid carcinoma	III	9	9

## Data Availability

Data sharing not applicable.
